# Catecholamines Attenuate LPS-Induced Inflammation through β2 Adrenergic Receptor Activation- and PKA Phosphorylation-Mediated TLR4 Downregulation in Macrophages

**DOI:** 10.3390/cimb46100675

**Published:** 2024-10-12

**Authors:** Cong Wang, Guo-Gang Feng, Junko Takagi, Yoshihiro Fujiwara, Tsuyoshi Sano, Hideaki Note

**Affiliations:** 1 Department of Gastroenterological Surgery, Aichi Medical University School of Medicine, 1-1 Yazakokarimata, Nagakute 480-1195, Aichi, Japan; ou.sou.008@mail.aichi-med-u.ac.jp (C.W.); sano.tsuyoshi.508@mail.aichi-med-u.ac.jp (T.S.); 2 Department of Anesthesiology, Aichi Medical University School of Medicine, 1-1 Yazakokarimata, Nagakute 480-1195, Aichi, Japan; note.hideaki.230@mail.aichi-med-u.ac.jp; 3 Division of Endocirnology and Metabolism, Department of Internal Medicine, Aichi Medical University School of Medicine, 1-1 Yazakokarimata, Nagakute 480-1195, Aichi, Japan; takagi.junko.946@mail.aichi-med-u.ac.jp; 4 Department of Anesthesiology and Pain Medicine, Fujita Health University Bantane Hospital, 3-6-10 Otobashi, Nakagawaku, Nagoya 454-8509, Aichi, Japan; yoshihiro.fujiwara@fujita-hu.ac.jp

**Keywords:** autonomic nervous system, catecholamine, β2 adrenergic receptor, lipopolysaccharide, TNFα, PKA phosphorylation, TLR4

## Abstract

Inflammation is a tightly regulated process involving immune receptor recognition, immune cell migration, inflammatory mediator secretion, and pathogen elimination, all essential for combating infection and restoring damaged tissue. However, excessive inflammatory responses drive various human diseases. The autonomic nervous system (ANS) is known to regulate inflammatory responses; however, the detailed mechanisms underlying this regulation remain incompletely understood. Herein, we aimed to study the anti-inflammatory effects and mechanism of action of the ANS in RAW264.7 cells. Quantitative PCR and immunoblotting assays were used to assess lipopolysaccharide (LPS)-induced tumor necrosis factor α (TNFα) expression. The anti-inflammatory effects of catecholamines (adrenaline, noradrenaline, and dopamine) and acetylcholine were examined in LPS-treated cells to identify the receptors involved. Catecholamines inhibited LPS-induced TNFα expression by activating the β2 adrenergic receptor (β2-AR). β2-AR activation in turn downregulated the expression of Toll-like receptor 4 (TLR4) by stimulating protein kinase A (PKA) phosphorylation, resulting in the suppression of TNFα levels. Collectively, our findings reveal a novel mechanism underlying the inhibitory effect of catecholamines on LPS-induced inflammatory responses, whereby β2-AR activation and PKA phosphorylation downregulate TLR4 expression in macrophages. These findings could provide valuable insights for the treatment of inflammatory diseases and anti-inflammatory drug development.

## 1. Introduction

Inflammation is an essential protective response activated by various stimuli and conditions, such as trauma, burns, and infections [1]. It is tightly regulated and involves the recognition of damaged cells and pathogens by immune receptors, ultimately resulting in the recruitment of immune cells, secretion of inflammatory cytokines, elimination of dead cells, and control of invading pathogens [2]. However, an excessive inflammatory response underpins the pathogenesis of numerous human diseases and, when particularly severe, can cause septic shock [3]. Therefore, exploring novel molecular mechanisms and therapeutic targets for the alleviation of excessive inflammatory responses remains crucial.

Lipopolysaccharide (LPS), an endotoxin released by Gram-negative bacteria, is a potent trigger of the inflammatory cascade [4,5]. LPS can elicit an excessive inflammatory response, resulting in microcirculatory dysfunction, multiple organ failure, and even death [6]. Therefore, LPS has been extensively explored and established as a crucial tool for inflammation research applied in in vivo as well as in vitro models [7,8]. Macrophages are the predominant type of immune cells residing in tissues, representing central components of the inflammatory response [9,10]. Upon activation by LPS, macrophages release a variety of inflammatory cytokines, leading to inflammation and potential toxicity [11,12]. Among the various cytokines, tumor necrosis factor α (TNFα) is required and sufficient for inflammation [13]. Additionally, pattern recognition receptors (PRRs) are essential elements of the innate immune system and trigger the inflammatory cascade [14]. Toll-like receptors (TLRs) were the first PRRs to be identified and have been studied extensively [15]. TLR4 responds to LPS, subsequently triggering a pro-inflammatory response that promotes the elimination of invading bacteria [14]. In contrast, uncontrolled and excessive activation of TLR4 by LPS may lead to sepsis, septic shock, or even death [16]. Hence, strict regulation of TLR4 activation and associated signaling is essential to maintain TLR-induced inflammatory responses at a balanced and appropriate level.

The autonomic nervous system (ANS), containing its sympathetic and parasympathetic branches (SNS and PNS, respectively), is crucial for the regulation and maintenance of physiological homeostasis. Many studies have shown that the SNS exerts anti-inflammatory effects by activating adrenergic receptors (ARs), especially β2-AR, expressed on immune cells [2,17,18]. While the SNS and PNS are considered functionally antagonistic, PNS activation was also shown to inhibit inflammation through the α7 nicotinic acetylcholine receptor (α7nAChR) [19]. These reports suggest that the ANS could affect immune cell function and regulate inflammatory responses; however, the associated regulatory mechanisms remain incompletely understood.

The ANS has been shown to regulate macrophage function through the release of neurotransmitters and activation of their cognate receptors, such as α7nAChR or β2-AR, expressed on macrophages [13,20]. RAW264.7, a mouse macrophage cell line, is an in vitro model commonly used for the study of LPS-induced inflammatory responses [3]. In the present study, we sought to evaluate the anti-inflammatory effects and regulatory mechanisms of the SNS and PNS in RAW264.7 cells. To this end, we characterized the anti-inflammatory effects of catecholamines and ACh, identifying the receptors involved in this response. Finally, we explored the relationship between β2-AR activation and TLR4 expression as well as the key factors involved. Our findings reveal a novel mechanism underlying the inhibitory effect of SNS on LPS-induced inflammatory responses, which provides valuable insights for the treatment of inflammatory diseases and anti-inflammatory drug development.

## 2. Materials and Methods

### 2.1. Materials

TRIzol reagent, Lipofectamine™ 3000, Lipofectamine™ RNAi MAX and the Pierce^TM^ BCA Protein Assay Kit were purchased from Thermo Fisher Scientific Inc. (Waltham, MA, USA). The pcDNA3.1 vector was obtained from Clontech (Palo Alto, CA, USA). Fetal bovine serum, penicillin, streptomycin, LPS (*Escherichia coli* O111:B4), adrenaline (Ad), noradrenaline (Nad), dopamine (DA), acetylcholine (ACh), phentolamine, propranolol, metoprolol, ICI 118,551, metaraminol, isoproterenol, dobutamine, fenoterol, and H89 were obtained from Sigma (St. Louis, MO, USA).

### 2.2. Cell Treatment

Murine macrophage RAW264.7 cells were obtained from the American Type Culture Collection (ATCC^®^ CRL-2278^TM^, Manassas, VA, USA) and cultured in Dulbecco’s Modified Eagle’s Medium (DMEM, Nacalai Tesque Inc., Kyoto, Japan) containing 10% heat-inactivated fetal bovine serum, 100 unit/mL penicillin, and 100 µg/mL streptomycin. Cells were maintained in a humidified 5% CO_2_ incubator at 37 °C.

RAW264.7 cells were treated with 1 μg/mL LPS as previously reported [21]. At 1, 2, 4, 6, 8, 10, or 12 h after treatment, total RNA and protein were extracted. TNFα mRNA and protein expression were examined. Ad (0.01–10 μM), Nad (0.01–10 μM), DA (0.1–100 μM), or ACh (0.01–10 μM) with or without LPS were added to cells and incubated for 2 h to investigate the effect of catecholamines and ACh on LPS-induced TNFα expression. In order to identify the receptors involved in LPS-induced TNFα expression, phentolamine (a non-selective α-AR antagonist, 0.1 μM), propranolol (a non-selective β-AR antagonist, 0.1 μM), metoprolol (a selective β1-AR antagonist, 0.1 μM), and ICI 118,551 (a selective β2-AR antagonist, 0.1 μM) were added to cells and incubated for 1 h. Then, LPS alone or in combination with catecholamines (Ad 0.1 μM, Nad 1 μM and DA 100 μM) was added and incubated for 2 h, whereafter TNFα expression was assessed.

Metaraminol (a non-selective α-AR agonist, 0.1 μM), isoproterenol (a non-selective β-AR agonist, 0.1 μM), dobutamine (a selective β1-AR agonist, 0.1 μM), or fenoterol (a selective β2-AR agonist, 0.1 μM) alone or in combination with LPS were added to cells and incubated for 2 h, whereafter TNFα expression was assessed. The concentrations of the agents used here were equal to or higher than those previously reported [22,23,24,25].

### 2.3. RT-qPCR

Total RNA was extracted from cells treated as described above using TRIzol™ Reagent. One microgram of total RNA was reverse-transcribed in the presence of random hexamers and oligo (dT) 20 primer using the ReverTra Ace qPCR RT kit (Toyobo, Osaka, Japan). A universal mouse reference RNA was used as the positive control (#740100, Thermo Fisher Scientific Inc.). Quantitative PCR (qPCR) was then performed using an Applied Biosystems^TM^ QuantStudio^TM^3 Real-Time PCR system, with the TaqMan^®^ Gene Expression Assay kit (Thermo Fisher Scientific Inc.). The qPCR reaction was executed following the standard cycling mode. The amplification conditions were held at 50 °C for 2 min and 95 °C for 10 min followed by 40 repeat cycles of denaturation at 95 °C for 15 s, and annealing and extension at 60 °C for 1 min. TNFα (ID: Mm00443258_m1), adrenergic receptor subtypes: α1-type (α1a, ID: Mm00442668_m1; α1b, ID: Mm00431685_m1; α1d, ID: Mm01328600_m1), α2-type (α2a, ID: Mm00845383_s1; α2b, ID: Mm00477390_s1; α2c, ID: Mm00431686_s1) and β-type (β1, ID: Mm00431701_s1; β2, ID: Mm02524224_s1; β3, ID: Mm02601819_g1), and glyceraldehyde-3-phosphate dehydrogenase (GAPDH, ID: Mm99999915_g1) expression were assessed. The cycle threshold (Ct) values, at which amplification entered the exponential phase were used as indicators of the relative amount of the initial target mRNA in each sample. The results are presented as ratios of the target mRNA levels to those of the internal control GAPDH as per the ΔΔCT method [26].

### 2.4. Immunoblotting Assay

RAW264.7 cells were homogenized in radioimmunoprecipitation assay lysis buffer (Nacalai Tesque Inc.) containing a mixture of protease inhibitor cocktail (Complete™ tables; Roche Applied Sciences, Mannheim, Germany). The lysates were sonicated and centrifuged at 1000× *g* for 10 min at 4 °C. The protein concentrations of samples were determined using a Pierce^TM^ BCA Protein Assay Kit. The same amount of sample was mixed with Laemmli denatured buffer, separated via sodium dodecyl sulfate-polyacrylamide gel electrophoresis (SDS-PAGE), and transferred to polyvinylidene fluoride (PVDF) membranes (Immobilon-P, Millipore, Billerica, MA, USA) [26]. Blots were detected with antibodies at a dilution of 1:1000 against TNFα (#11948), protein kinase A (PKA, #4782), phospho-PKA (p-PKA, #5661), TLR4 (#14358), and GAPDH (#5174), followed by horseradish peroxidase-conjugated secondary antibodies (#7074 and #7076, 1:2000). These antibodies were obtained from Cell Signaling Technology (Danvers, MA, USA). In addition, some membranes were probed with an antibody against β2-AR (1:500, sc-81577, Santa Cruz Biotechnology, Santa Cruz, CA, USA). Proteins were visualized using ECL Select^TM^ Western Blotting Reagent (Cytiva, Tokyo, Japan). GAPDH was used as an internal control to verify equivalent loading across samples. Band densities were quantitatively analyzed using ImageJ software 1.54g (National Institutes of Health).

### 2.5. siRNA Transfection and β2-AR Knockdown

Silencer Select Pre-designed siRNAs for β2-AR (β2-AR-siRNA, ID: s62080) and a control siRNA (Con-siRNA, catalog #4390843) were purchased from Thermo Fisher Scientific Inc. RAW264.7 cells were transfected with 5 nM β2-AR-siRNA and Con-siRNA using Lipofectamine RNAiMAX according to the manufacturer’s protocol [26]. Eighteen hours after transfection, total protein was extracted, and β2-AR and TLR4 expression was examined. In order to evaluate the effect of β2-AR knockdown on LPS-induced TNFα expression, LPS, alone or in combination with Ad, was added to cells and incubated for 2 h. TNFα expression was then determined.

### 2.6. Construction of β2-AR-Overexpressing Plasmid and Transient Overexpression

The entire coding region of mouse β2-AR was amplified via PCR and inserted into the Not I-EcoR I restriction sites of the pcDNA 3.1 (pcDNA) plasmid to construct a β2-AR overexpressing plasmid (pcDNA-β2-AR). This construct was further confirmed through sequencing. RAW264.7 cells were transfected with either the pcDNA-β2-AR plasmid or a control (pcDNA) plasmid using Lipofectamine 3000 [26]. After 8 h of transfection, the medium was replaced with supplemented DMEM, and cells were cultured for 10 h. β2-AR expression was then examined to confirm the effect of plasmid transfection.

Eighteen hours after transfection, LPS, alone or in combination with Ad, was added to the cells and incubated for 2 h. TNFα expression was then determined. Alternatively, 8 h after transfection, the medium was replaced with supplemented DMEM with or without H89 (a potent and selective inhibitor of cyclic AMP-dependent PKA, 10 μM) for 10 h. Ad was then added and incubated for 2 h. TLR4, PKA, and p-PKA levels were determined via immunoblotting.

### 2.7. Statistical Analysis

Results are expressed as means ± standard error (SE). For multiple comparisons, a one-way repeated-measures analysis of variance was performed with Scheffe or Bonferroni corrections. Two-tailed Student’s *t*-tests were used for comparisons between the two groups. Statistical analyses were performed using GraphPad Prism 6 (San Diego, CA, USA). *p* < 0.05 was considered significant.

## 3. Results

### 3.1. LPS Induced TNFα Expression in RAW264.7 Cells

**Figure 1:**
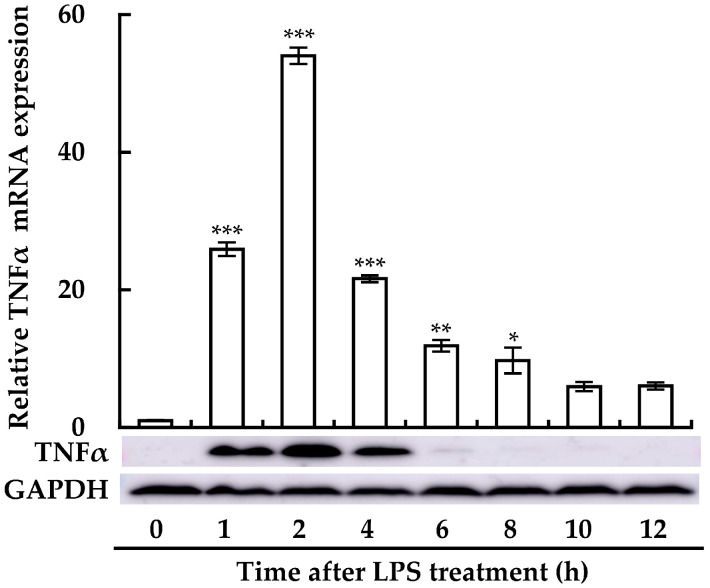
Time-dependent effect of LPS on TNFα production in RAW264.7 cells. TNFα expression was measured at the mRNA level (upper panel) via RT-qPCR and at the protein level (lower panel) through immunoblotting, following treatment with 1 μg/mL LPS at various time points (0–12 h). Data from qPCR analyses are expressed as mean ± SE (*n* = 3). * *p* < 0.05, ** *p* < 0.01, and *** *p* < 0.001 vs. without LPS treatment (0 time). For the immunoblotting, one representative result from three independent experiments is shown. GAPDH was used as a loading control.

We first examined the expression of TNFα in LPS-treated RAW264.7 cells using RT-qPCR and immunoblotting assays. TNFα expression was clearly enhanced from 1 h after LPS treatment, with a peak at 2 h, then gradually diminishing at both the mRNA and protein levels (Figure 1). These results indicated that LPS induces an inflammatory response in macrophages in a time-dependent manner. As LPS-induced TNFα expression was the highest at 2 h after treatment with LPS, the following experiments were carried out at this time point.

### 3.2. Ad, Nad, and DA Suppressed LPS-Induced TNFα Expression

**Figure 2:**
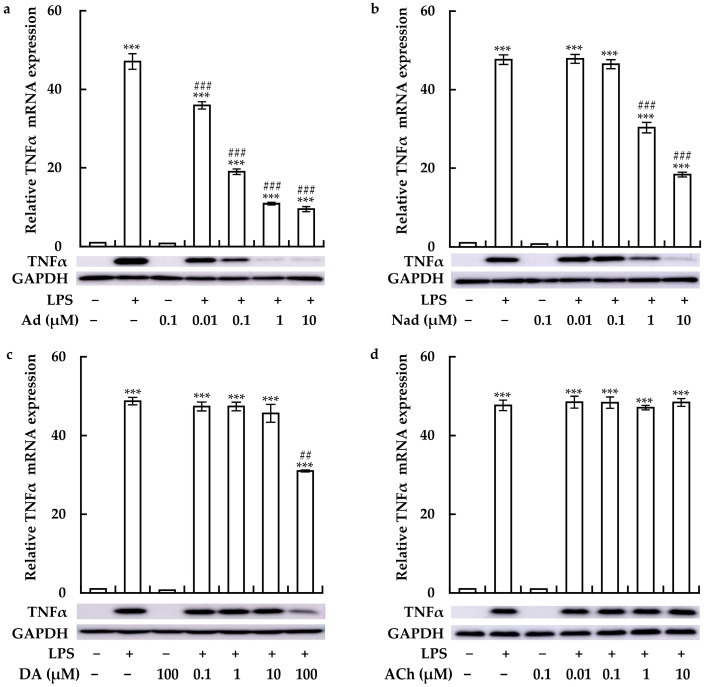
Concentration-dependent effects of adrenaline (Ad), noradrenaline (Nad), dopamine (DA), and acetylcholine (ACh) on LPS-induced TNFα production in RAW264.7 cells. Cells were treated with varying concentrations of (**a**) Ad (0.01–10 μM), (**b**) Nad (0.01–10 μM), (**c**) DA (0.1–100 μM), and (**d**) ACh (0.01–10 μM), in the presence or absence of 1 μg/mL LPS for 2 h. TNFα expression was assessed at both the mRNA level (upper panel) via RT-qPCR and the protein level (lower panel) via immunoblotting. Data from qPCR analyses are expressed as mean ± SE (*n* = 3). *** *p* < 0.001 vs. without any treatment; ## *p* < 0.01 and ### *p* < 0.001 vs. with LPS treatment. For the immunoblotting, one representative result from three independent experiments is shown. GAPDH was used as a loading control.

We then examined the effects of Ad, Nad, DA, and ACh on LPS-induced TNFα expression. Ad, Nad, and DA clearly suppressed LPS-induced TNFα expression at the mRNA and protein levels in a concentration-dependent manner (Figure 2a–c). Notably, compared to Nad and DA, Ad exhibited a stronger inhibitory effect on LPS-induced TNFα expression. In contrast, treatment with ACh failed to affect TNFα expression (Figure 2d). These observations suggested that the SNS may suppress LPS-induced inflammatory response in macrophages. Lower concentrations of Ad (0.1 μM), Nad (1 μM), and DA (100 μM) markedly inhibited the LPS-induced TNFα expression and were thus, used in the subsequent experiments.

### 3.3. Ad, Nad, and DA Suppressed LPS-Induced TNFα Expression by Activating β2-AR

**Figure 3:**
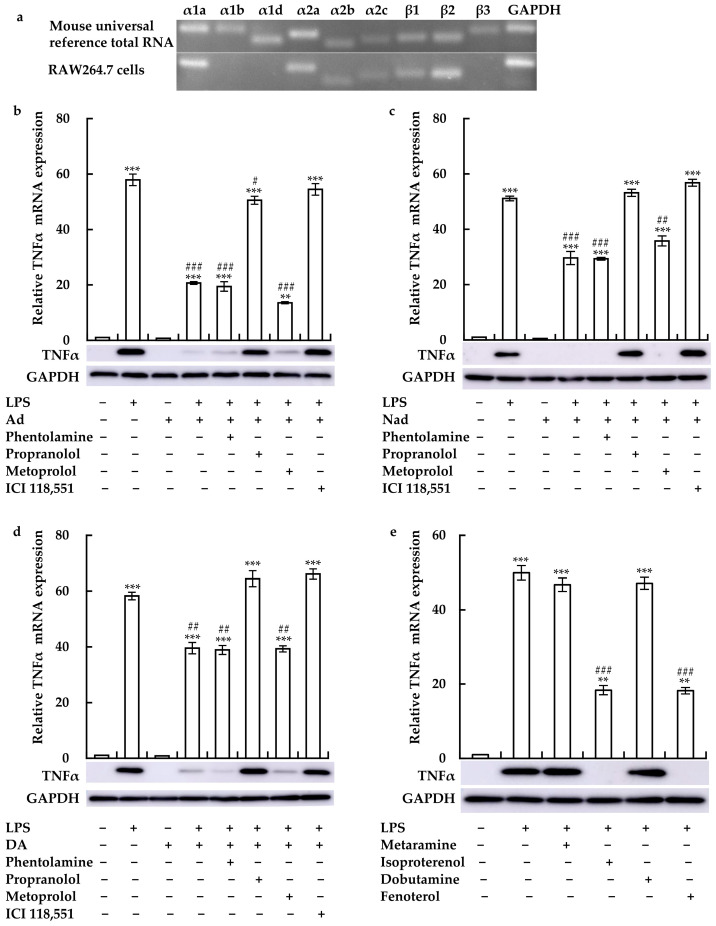
Roles of the β2 adrenergic receptor (β2-AR) in the effects of Ad, Nad, and DA on TNFα expression in LPS-treated RAW264.7 cells. (**a**) AR expression in RAW 264.7 cells. Effects of phentolamine, propranolol, metoprolol, or ICI 118,551 on TNFα expression in cells treated with LPS with or without (**b**) Ad, (**c**) Nad, and (**d**) DA. (**e**) Cells were also treated with LPS with or without metaramine, isoproterenol, dobutamine, or fenoterol. Data from qPCR analyses are expressed as means ± SE (*n* = 3). ** *p* < 0.01, and *** *p* < 0.001 vs. without any treatment; # *p* < 0.05, ## *p* < 0.01 and ### *p* < 0.001 vs. with LPS treatment. For the immunoblotting, one representative result from three independent experiments is shown. GAPDH was used as a loading control.

To identify the receptor through which catecholamines regulated LPS-induced TNFα expression, we examined AR expression using RT-qPCR. Specifically, α1a-, α1b-, α1d-, α2a-, α2b-, α2c-, β1-, and β2-AR mRNA expression was detected in RAW264.7 cells (Figure 3a). Pre-treatment with propranolol and ICI 118,551 noticeably attenuated the inhibitory effects of Ad, Nad, and DA on LPS-induced TNFα expression (Figure 3b–d). However, phentolamine and metoprolol failed to do so. Furthermore, we examined the effects of AR agonists on LPS-induced TNFα expression. As expected, treatment with isoproterenol and fenoterol also considerably suppressed LPS-induced TNFα expression, while metaraminol and dobutamine did not work (Figure 3e). These findings indicated that the SNS may suppress LPS-induced inflammatory response in macrophages via β2-AR activation.

### 3.4. β2-AR Mediated the Inhibitory Effect of Ad on LPS-Induced TNFα Expression

**Figure 4:**
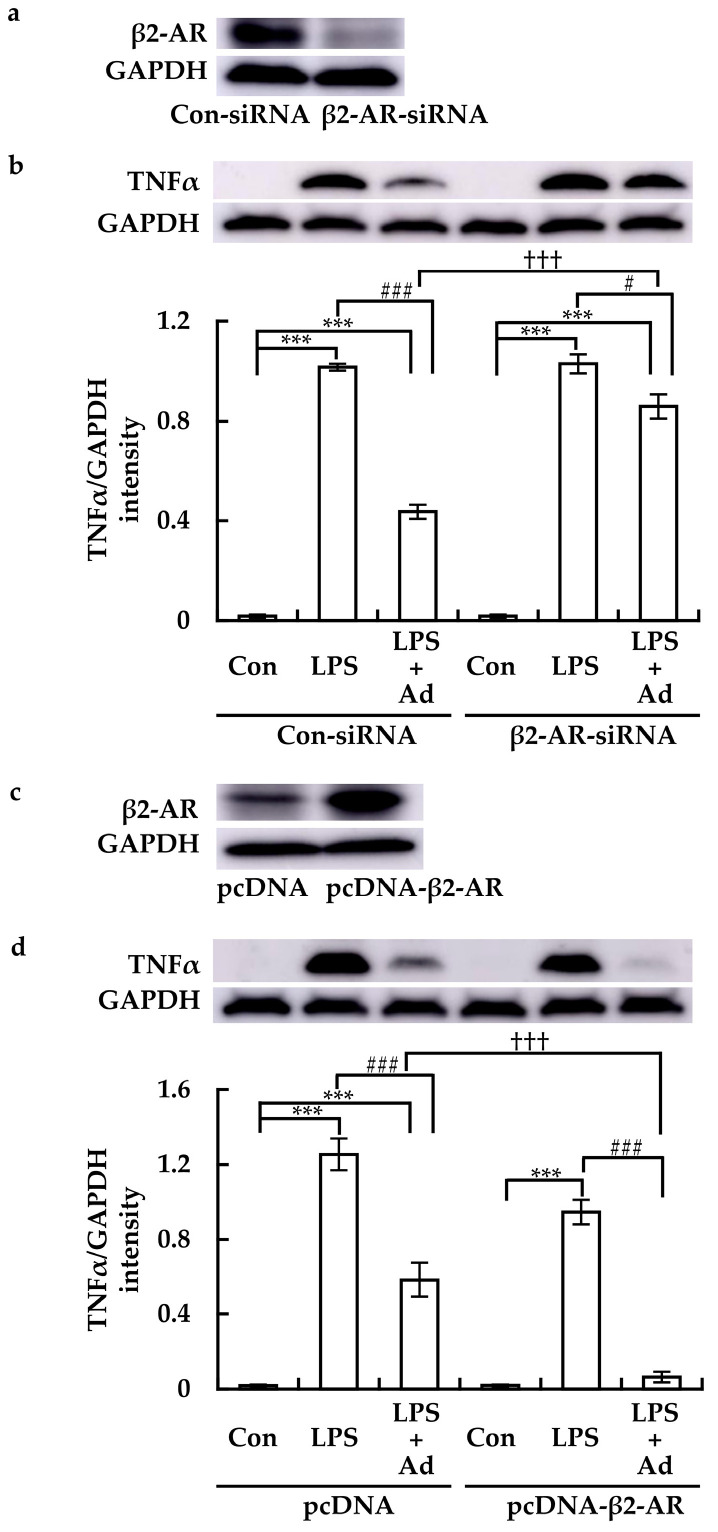
Effects of β2-AR knockdown or overexpression on the expression of TNFα in LPS-treated RAW264.7 cells treated with Ad. Cells were transfected with the specific siRNA targeting β2-AR (β2-AR-siRNA) and control siRNA (Con-siRNA), and plasmid overexpressing β2-AR (pcDNA-β2-AR) and its control (pcDNA), respectively. (**a**,**c**) β2-AR expression was then examined via immunoblotting. (**b**,**d**) Transfected cells were treated with LPS with or without Ad for 2 h, and TNFα protein levels were determined (upper panel). One representative result from three independent experiments is shown. GAPDH was used as a loading control. The band density was quantitatively analyzed using ImageJ software. The results (lower panel in (**b**,**d**)) are expressed as mean ± SE (*n* = 3). *** *p* < 0.001; # *p* < 0.05 and ### *p* < 0.001; ††† *p* < 0.001.

Compared to Con-siRNA transfection, β2-AR-siRNA transfection potently suppressed β2-AR protein levels in RAW264.7 cells (Figure 4a). In Con-siRNA-transfected cells, LPS clearly induced TNFα expression, and this effect was suppressed by Ad (Figure 4b). Although β2-AR-siRNA did not affect LPS-induced TNFα expression on its own, it clearly reduced the inhibitory effect of Ad (Figure 4b).

In contrast to transfection with pcDNA, transfection with pcDNA-β2-AR elicited a clear transient increase in β2-AR expression (Figure 4c). In pcDNA-transfected cells, Ad potently suppressed LPS-induced TNFα expression (Figure 4d). Compared to pcDNA transfection, pcDNA-β2-AR transfection slightly suppressed LPS-induced TNFα expression, enhancing the inhibitory effect of Ad (Figure 4d). These results further confirmed that β2-AR is involved in SNS-mediated suppression of LPS-induced inflammatory response in macrophages.

### 3.5. β2-AR Regulated TLR4 Receptor Expression by Stimulating PKA Phosphorylation

**Figure 5:**
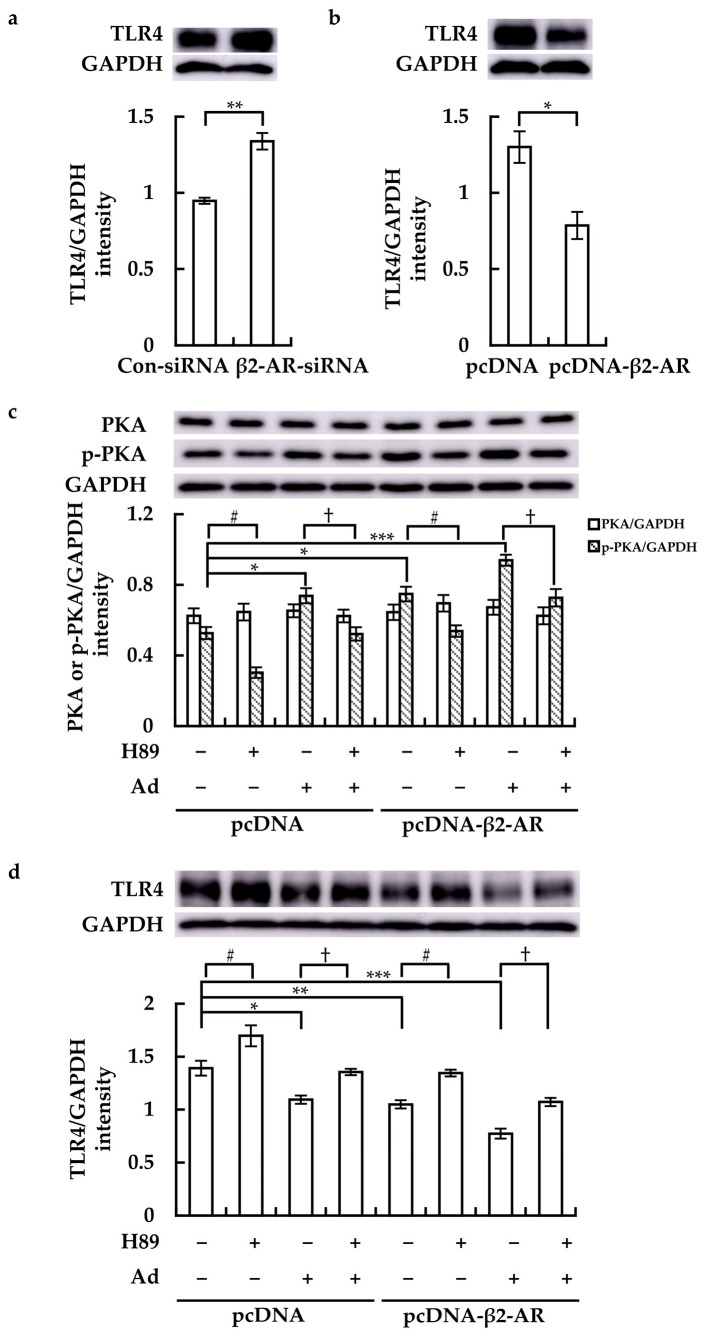
Roles of β2-AR activation in PKA phosphorylation (p-PKA) and TLR4 expression in RAW264.7 cells. (**a**,**b**) Cells were transfected as described above, and TLR4 protein levels were assessed. After transfection with pcDNA or pcDNA-β2-AR for 8 h, H89 was added and incubated for 10 h. Ad was then added and incubated for 2 h. PKA, p-PKA, and TLR4 protein levels were detected (upper panel in (**c**,**d**)). One representative result from three independent experiments is shown. GAPDH was used as a loading control. Band density was quantitatively analyzed using ImageJ software, and the results (lower panel) are expressed as means ± SE (*n* = 3). * *p* < 0.05, ** *p* < 0.01, and *** *p* < 0.001; # *p* < 0.05; † *p* < 0.05.

Compared to Con-siRNA transfection, β2-AR-siRNA transfection obviously enhanced TLR4 expression in RAW264.7 cells (Figure 5a). Meanwhile, pcDNA-β2-AR transfection potently suppressed TLR4 expression (Figure 5b,d). While transfection with pcDNA-β2-AR did not affect PKA protein level, it clearly enhanced PKA phosphorylation (p-PKA), which was further increased by Ad treatment. Whereas H89 not only obviously suppressed PKA phosphorylation but also impeded the Ad-induced PKA phosphorylation (Figure 5c). Compared to cells transfected with pcDNA, in which Ad clearly downregulated TLR4, Ad exhibited a stronger inhibitory effect on TLR4 expression in pcDNA-β2-AR-transfected cells (Figure 5d). Meanwhile, H89 not only enhanced TLR4 expression but also attenuated the suppressive effect of Ad (Figure 5d). These findings indicated that activation of β2-AR could downregulate the expression of TLR4 by stimulating PKA phosphorylation.

## 4. Discussion

Inflammation is the key response driven by the immune system and may be activated by various factors, such as pathogens, harmful substances, and irradiation [27]. It is well-established that a controlled inflammatory response is conducive to broad-spectrum protection against infection. In contrast, chronic and uncontrolled inflammatory responses often cause severe tissue damage, culminating in multi-organ failure with high mortality rates [3]. Immune regulation is thus critical, with the ANS emerging as a modulator of immune responses through the release of sympathetic and parasympathetic neurotransmitters and neuropeptides [28,29].

In the present study, we demonstrated that LPS induces an inflammatory response in RAW264.7 cells, which is mediated by catecholamines. Our findings are consistent with those of previous studies, which demonstrated that catecholamines could decrease LPS-induced cytokine production in vitro and in vivo [30,31]. In patients with sepsis and septic shock, Nad and Ad (in severe cases) are routinely administered as potent vasopressors for the treatment of hypotension and concomitant organ hypoperfusion [28]. Similarly, our results suggest that the administration of catecholamines in emergency situations not only stabilizes circulatory dynamics but also suppresses the inflammatory response. Expression of α7nAChR in RAW264.7 cells has been reported previously [13]. However, treatment with ACh did not affect LPS-induced TNFα expression in our study. This finding suggested that the SNS may regulate LPS-induced inflammation through neurotransmitter release and signaling via cognate receptors in macrophages. Martelli et al. also reported that the SNS can regulate inflammation in LPS-challenged rats [32]. In addition, previous studies indicated that activated macrophages and RAW264.7 cells can produce catecholamines [33,34]. Moreover, plasma catecholamines levels can increase up to 20-fold (2–8 × 10^−8^ M) during severe stress and sepsis in animal models [35]. Thus, these responses may be potentially involved in the anti-inflammatory effects of the SNS.

Next, we investigated the receptors through which catecholamines regulate LPS-induced TNFα expression. Our results indicated that β2-AR, but not α- or β1-AR, is implicated in the anti-inflammatory effect of catecholamines. These findings are consistent with those of previous studies showing that β2-AR activation suppresses the LPS-induced inflammatory response [36,37]. β2-ARs exist in airway smooth muscles, cardiac muscles, uterine muscles, alveolar type II cells, vascular endothelium, and immune cells [38]. Numerous studies reported a cardioprotective role for β2-AR activation in heart failure, which attenuates the positive inotropic effects of β1-AR stimulation and activates cardioprotective signaling pathways [39,40]. Taken together, these results suggest that activation of β2-AR not only suppresses inflammatory responses but also has potential protective effects on cardiomyocytes. Although the α2-AR agonist dexmedetomidine was recently reported to have an anti-inflammatory property and alleviate pulmonary edema in LPS-induced acute lung injury in rats and alveolar epithelial cell injury in A549 cells [41], we did not observe any anti-inflammatory effects of metaraminol in our study. Thus, future studies should explore the roles of other adrenergic receptor subtypes, such as α2-AR, in the anti-inflammatory effects of catecholamines using different inflammatory models. In addition, compared to Nad and DA, Ad elicited a stronger anti-inflammatory effect, which may be due to its higher affinity for β2-AR. Therefore, we employed Ad as a representative catecholamine to investigate the effect of β2-AR activation on LPS-induced inflammation. We found that knockdown or overexpression of β2-AR affected the suppressive effect of Ad. Thus, these results further confirmed that catecholamines exert their anti-inflammatory effects through β2-AR activation in macrophages.

TLR4 plays a central role in the occurrence and development of various inflammatory diseases [14]. It binds to LPS and triggers downstream inflammatory cascades, potentially leading to the excessive release of inflammatory factors [42]. Previous studies have shown that TLR4 silencing could effectively suppress LPS-induced inflammatory responses [43]. Thus, TLR4 represents a potential therapeutic target for various inflammatory diseases. However, the relationship between catecholamines and TLR4 expression remains unclear. Therefore, we investigated whether catecholamines regulate TLR4 expression through β2-AR activation in RAW264.7 cells. Our results showed that β2-AR activation could negatively regulate TLR4 expression. We then investigated the underlying mechanism through which β2-AR regulates TLR4 expression. Previous studies have reported that β2-AR exhibits an anti-inflammatory effect by phosphorylating PKA [44]. Therefore, we investigated the effect of PKA phosphorylation on TLR4 expression. Our results showed that activation of β2-AR stimulated PKA phosphorylation, which consequently downregulated TLR4 expression. These findings indicate that PKA may be a key mediator of the anti-inflammatory effect of catecholamines. Our observations are consistent with a previous report which found that immune complexes could reduce TLR4 expression via activation of the cAMP/PKA pathway, thereby inhibiting TNFα and IL-6 expression in macrophages [45]. In addition, it has been reported that TLR4 is also involved in the development of chronic inflammation [14]. Taken together, these results suggest that β2-AR activation may play an important role in regulating chronic inflammatory responses through suppressing TLR4 expression and contribute to the treatment of chronic inflammatory diseases. Nevertheless, these effects should be confirmed in future studies. Although we uncovered a novel mechanism underlying the anti-inflammatory effects of ANS, a limitation of this study is that this mechanism was demonstrated only in RAW264.7 cells. Therefore, further studies using peritoneal or tissue macrophages, and animal models are required to validate our results.

In summary, we demonstrated that catecholamines suppress LPS-induced TLR4-driven inflammation in macrophages through the activation of β2-AR. Further, we propose a novel mechanism whereby β2-AR activation negatively regulates TLR4 by stimulating PKA phosphorylation to ultimately suppress the production of inflammatory factors. Our results suggest that SNS could regulate the inflammatory response, at least in part, via β2-AR/PKA-mediated TLR4 downregulation in macrophages. These findings provide novel insights into the regulatory function of the ANS during inflammatory responses, which are of great relevance for the treatment of inflammatory diseases and anti-inflammatory drug development.

## Data Availability

The original contributions presented in the study are included in the article, further inquiries can be directed to the corresponding author.

## References

[1] Medzhitov R. (2008). Origin and physiological roles of inflammation. Nature.

[2] Ağaç D., Estrada L.D., Maples R., Hooper L.V., Farrar J.D. (2018). The β2-adrenergic receptor controls inflammation by driving rapid IL-10 secretion. Brain Behav. Immun..

[3] Wang S., Liu F., Tan K.S., Ser H.L., Tan L.T., Lee L.H., Tan W. (2020). Effect of (R)-salbutamol on the switch of phenotype and metabolic pattern in LPS-induced macrophage cells. J. Cell Mol. Med..

[4] Tanaka S., Couret D., Tran-Dinh A., Duranteau J., Montravers P., Schwendeman A., Meilhac O. (2020). High-density lipoproteins during sepsis: From bench to bedside. Crit. Care.

[5] Ioanna Z., Katerina B., Irene A. (2021). Immunotherapy-on-chip against an experimental sepsis model. Inflammation.

[6] Caffaratti C., Plazy C., Mery G., Tidjani A.R., Fiorini F., Thiroux S., Toussaint B., Hannani D., Le Gouellec A. (2021). What we know so far about the metabolite-mediated microbiota-intestinal immunity dialogue and how to hear the sound of this crosstalk. Metabolites.

[7] Pussinen P.J., Kopra E., Pietiainen M., Lehto M., Zaric S., Paju S., Salminen A. (2022). Periodontitis and cardiometabolic disorders: The role of lipopolysaccharide and endotoxemia. Periodontol. 2000.

[8] Venkataranganayaka Abhilasha K., Kedihithlu M.G. (2021). Bacterial lipoproteins in sepsis. Immunobiology.

[9] Oberholzer A., Oberholzer C., Moldawer L.L. (2001). Sepsis syndromes: Understanding the role of innate and acquired immunity. Shock.

[10] Beutler B., Poltorak A. (2001). Sepsis and evolution of the innate immune response. Crit. Care Med..

[11] Tisoncik J.R., Korth M.J., Simmons C.P., Farrar J., Martin T.R., Katze M.G. (2012). Into the eye of the cytokine storm. Microbiol. Mol. Biol. Rev..

[12] Feng J., Liu L., He Y., Wang M., Zhou D., Wang J. (2021). Novel insights into the pathogenesis of virus-induced ARDS: Review on the central role of the epithelial-endothelial barrier. Expert. Rev. Clin. Immunol..

[13] Tracey K.J. (2002). The inflammatory reflex. Nature.

[14] Ciesielska A., Matyjek M., Kwiatkowska K. (2021). TLR4 and CD14 trafficking and its influence on LPS-induced pro-inflammatory signaling. Cell Mol. Life Sci..

[15] Yang Y., Lv J., Jiang S., Ma Z., Wang D., Hu W., Deng C., Fan C., Di S., Sun Y. (2016). The emerging role of Toll-like receptor 4 in myocardial inflammation. Cell Death Dis..

[16] Poltorak A., He X., Smirnova I., Liu M.Y., Van Huffel C., Du X., Birdwell D., Alejos E., Silva M., Galanos C. (1998). Defective LPS signaling in C3H/HeJ and C57BL/10ScCr mice: Mutations in Tlr4 gene. Science.

[17] Umene R., Nakamura Y., Wu C.H., Muta K., Nishino T., Inoue T. (2023). Induction of tetraspanin 13 contributes to the synergistic anti-inflammatory effects of parasympathetic and sympathetic stimulation in macrophages. Biochem. Biophys. Res. Commun..

[18] Pongratz G., Straub R.H. (2014). The sympathetic nervous response in inflammation. Arthritis Res. Ther..

[19] Zhu S., Huang S., Xia G., Wu J., Shen Y., Wang Y., Ostrom R.S., Du A., Shen C., Xu C. (2021). Anti-inflammatory effects of α7-nicotinic ACh receptors are exerted through interactions with adenylyl cyclase-6. Br. J. Pharmacol..

[20] Izeboud C.A., Mocking J.A., Monshouwer M., van Miert A.S., Witkamp R.F. (1999). Participation of beta-adrenergic receptors on macrophages in modulation of LPS-induced cytokine release. J. Recept. Signal Transduct. Res..

[21] Kang J.K., Kang H.K., Hyun C.G. (2022). Anti-Inflammatory Effects of Spiramycin in LPS-Activated RAW 264.7 Macrophages. Molecules.

[22] Huang J.L., Zhang Y.L., Wang C.C., Zhou J.R., Ma Q., Wang X., Shen X.H., Jiang C.L. (2012). Enhanced phosphorylation of MAPKs by NE promotes TNF-α production by macrophage through α adrenergic receptor. Inflammation.

[23] Salameh A., Blanke K., Dhein S., Janousek J. (2010). Cardiac gap junction channels are upregulated by metoprolol: An unexpected effect of beta-blockers. Pharmacology.

[24] Ardestani P.M., Evans A.K., Yi B., Nguyen T., Coutellier L., Shamloo M. (2017). Modulation of neuroinflammation and pathology in the 5XFAD mouse model of Alzheimer’s disease using a biased and selective beta-1 adrenergic receptor partial agonist. Neuropharmacology.

[25] Faisy C., Grassin-Delyle S., Blouquit-Laye S., Brollo M., Naline E., Chapelier A., Devillier P. (2014). Wnt/β-catenin signaling modulates human airway sensitization induced by β2-adrenoceptor stimulation. PLoS ONE.

[26] Feng G.G., Li C., Huang L., Tsunekawa K., Sato Y., Fujiwara Y., Komatsu T., Honda T., Fan J.H., Goto H. (2010). Naofen, a novel WD40-repeat protein, mediates spontaneous and tumor necrosis factor-induced apoptosis. Biochem. Biophys. Res. Commun..

[27] Chen L., Deng H., Cui H., Fang J., Zuo Z., Deng J., Li Y., Wang X., Zhao L. (2017). Inflammatory responses and inflammation-associated diseases in organs. Oncotarget.

[28] Roewe J., Higer M., Riehl D.R., Gericke A., Radsak M.P., Bosmann M. (2017). Neuroendocrine Modulation of IL-27 in Macrophages. J. Immunol..

[29] Sternberg E.M. (2006). Neural regulation of innate immunity: A coordinated nonspecific host response to pathogens. Nat. Rev. Immunol..

[30] Merrill K.M., Hull M.B., Stoker A., DeClue A.E. (2021). In vitro effects of epinephrine, norepinephrine, and dobutamine on lipopolysaccharide-stimulated production of tumor necrosis factor-α, interleukin-6, and interleukin-10 in blood from healthy dogs. Am. J. Vet. Res..

[31] Yoshioka Y., Sugino Y., Shibagaki F., Yamamuro A., Ishimaru Y., Maeda S. (2020). Dopamine attenuates lipopolysaccharide-induced expression of proinflammatory cytokines by inhibiting the nuclear translocation of NF-κB p65 through the formation of dopamine quinone in microglia. Eur. J. Pharmacol..

[32] Martelli D., Yao S.T., McKinley M.J., McAllen R.M. (2014). Reflex control of inflammation by sympathetic nerves, not the vagus. J. Physiol..

[33] Brown S.W., Meyers R.T., Brennan K.M., Rumble J.M., Narasimhachari N., Perozzi E.F., Ryan J.J., Stewart J.K., Fischer-Stenger K. (2003). Catecholamines in a macrophage cell line. J. Neuroimmunol..

[34] Nguyen K.D., Qiu Y., Cui X., Goh Y.P., Mwangi J., David T., Mukundan L., Brombacher F., Locksley R.M., Chawla A. (2011). Alternatively activated macrophages produce catecholamines to sustain adaptive thermogenesis. Nature.

[35] Oberbeck R., Schmitz D., Wilsenack K., Schüler M., Pehle B., Schedlowski M., Exton M.S. (2004). Adrenergic modulation of survival and cellular immune functions during polymicrobial sepsis. Neuroimmunomodulation.

[36] Ryan K.J., Griffin É., Yssel J.D., Ryan K.M., McNamee E.N., Harkin A., Connor T.J. (2013). Stimulation of central β2-adrenoceptors suppresses NFκB activity in rat brain: A role for IκB. Neurochem. Int..

[37] Hasegawa S., Inoue T., Nakamura Y., Fukaya D., Uni R., Wu C.H., Fujii R., Peerapanyasut W., Taguchi A., Kohro T. (2021). Activation of Sympathetic Signaling in Macrophages Blocks Systemic Inflammation and Protects against Renal Ischemia-Reperfusion Injury. J. Am. Soc. Nephrol..

[38] Motiejunaite J., Amar L., Vidal-Petiot E. (2021). Adrenergic receptors and cardiovascular effects of catecholamines. Ann Endocrinol.

[39] Baker A.J. (2014). Adrenergic signaling in heart failure: A balance of toxic and protective effects. Pflugers Arch..

[40] Chesley A., Lundberg M.S., Asai T., Xiao R.P., Ohtani S., Lakatta E.G., Crow M.T. (2000). The beta(2)-adrenergic receptor delivers an antiapoptotic signal to cardiac myocytes through G(i)-dependent coupling to phosphatidylinositol 3′-kinase. Circ. Res..

[41] Jiang Y., Xia M., Xu J., Huang Q., Dai Z., Zhang X. (2021). Dexmedetomidine alleviates pulmonary edema through the epithelial sodium channel (ENaC) via the PI3K/Akt/Nedd4-2 pathway in LPS-induced acute lung injury. Immunol. Res..

[42] Cao C., Chai Y., Shou S., Wang J., Huang Y., Ma T. (2018). Toll-like receptor 4 deficiency increases resistance in sepsis-induced immune dysfunction. Int. Immunopharmacol..

[43] Hu X., Zhou W., Wu S., Wang R., Luan Z., Geng X., Xu N., Zhang Z., Ruan Z., Wang Z. (2022). Tacrolimus alleviates LPS-induced AKI by inhibiting TLR4/MyD88/NF-κB signalling in mice. J. Cell Mol. Med..

[44] Yanagawa Y., Matsumoto M., Togashi H. (2011). Adrenoceptor-mediated enhancement of interleukin-33 production by dendritic cells. Brain Behav. Immun..

[45] Zhang Y., Liu S., Liu J., Zhang T., Shen Q., Yu Y., Cao X. (2009). Immune complex/Ig negatively regulate TLR4-triggered inflam-matory response in macrophages through Fc gamma RIIb-dependent PGE2 production. J. Immunol..

